# Gut microbiota-derived cholic acid ameliorates lung inflammation in bronchopulmonary dysplasia through modulation of macrophage function

**DOI:** 10.1016/j.isci.2026.115398

**Published:** 2026-03-17

**Authors:** Dongying Zhao, Caixia Gao, Danying Zhu, Xiaoyan Zheng, Jiping Sun, Chengbo Liu, Lei Chen, Lei Shen, Xingyun Wang, Yongjun Zhang

**Affiliations:** 1 Department of Pediatrics, Xinhua Hospital, Shanghai Jiao Tong University School of Medicine, 1665 Kong Jiang Road, Shanghai 200092, China; 2 Precision Research Center for Refractory Diseases, Shanghai General Hospital, Shanghai Jiao Tong University School of Medicine, Shanghai 201610, China; 3 Department of Pulmonary, Shanghai Children’s Hospital, School of Medicine, Shanghai Jiao Tong University, Shanghai 200062, China; 4 Shanghai Institute of Immunology, Shanghai Jiao Tong University School of Medicine, Shanghai 200025, China; 5 Renji Hospital Affiliated with Shanghai Jiao Tong University School of Medicine, Shanghai 200127, China; 6 Hongqiao International Institute of Medicine, Tongren Hospital, Shanghai Jiao Tong University School of Medicine, Shanghai 200005, China

**Keywords:** pediatrics, microbiome, metabolomics

## Abstract

This study explores the impact of gut microbiota-derived metabolites on the pathogenesis of bronchopulmonary dysplasia (BPD), focusing on their roles in macrophage plasticity and inflammation. In a prospective nested case-control cohort of 30 infants with BPD and 33 preterm controls, 16S ribosomal RNA (16S rRNA) and mass spectrometry analyses identified seven differential gut bacterial genera, with depleted *Streptococcus* and enriched *Klebsiella* in patients with BPD, alongside reduced fecal and serum cholic acid levels. In chorioamnionitis-induced rat models of BPD, cholic acid supplementation alleviated lung inflammation by regulating macrophage migration and polarization. RNA-sequencing and *in vitro* experiments revealed that cholic acid acts by inhibiting hypoxia-inducible factor-1α (HIF-1α) expression and transcriptional activity, an effect that was abolished by HIF-1α silencing. These findings connect the gut microbiota to BPD, highlighting cholic acid as a key regulator of macrophage function through the HIF-1α pathway in mitigating inflammation and providing new clues for understanding and intervening in BPD.

## Highlights

• Intestinal dysbiosis in BPD is associated with reduced levels of the microbial metabolite CA
• CA ameliorates BPD lung inflammation by regulating macrophage function via HIF-1α pathway
• We identify a gut microbiota-CA-macrophage axis as a novel therapeutic target for BPD

## Graphical abstract

**Figure:**
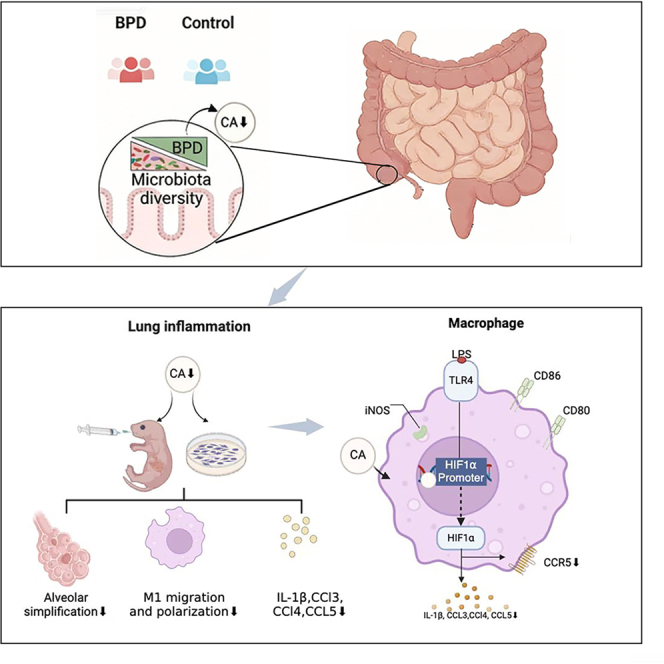

## Introduction

Bronchopulmonary dysplasia (BPD) is a common complication among preterm infants.^1^ Despite notable progress in neonatal care for preterm deliveries, the prevalence of BPD has shown no significant decline.^2^ This disorder imposes lifelong consequences, including neurodevelopmental impairments, pulmonary hypertension, and sustained tracheobronchomalacia into adulthood.^3^^,^^4^

Although tracheomalacia and bronchomalacia may develop in BPD, its hallmark feature is disrupted perinatal lung development, specifically arrested alveolarization and vascularization, resulting in fewer alveoli, enlarged airspaces, and abnormal vascular remodeling. These structural abnormalities drive gas exchange inefficiency, progressive pulmonary hypertension, and heightened susceptibility to infection, manifesting as chronic respiratory distress, exercise intolerance, and right ventricular hypertrophy.^5^

Currently, the pathophysiology of BPD involves preterm-related immaturity, mechanical ventilation-induced barotrauma, hyperoxia, and intrauterine or postnatal inflammation, including chorioamnionitis and sepsis, which collectively disrupt the balance between lung growth and repair.^6^ Recent evidence highlights a significant correlation between exposure to inflammatory stressors, such as chorioamnionitis, early-onset sepsis, and late-onset sepsis, and the progression of BPD.^7^^,^^8^ The microbiota-mediated gut-lung axis has been identified as a contributing factor to lung inflammation.^9^^,^^10^ However, the causal relationship between microbial metabolites and the progression of BPD remains unclear.

Metabolites derived from the gut microbiota, such as bile acids (BAs) and short-chain fatty acids (SCFAs), have demonstrated the ability to mitigate inflammatory and immune responses, cytokine production, mucus metaplasia, and airway hyperresponsiveness in allergic airway diseases.^9^ Our prior work revealed significantly altered bile acid profiles in neonates with cholestasis compared with healthy infants.^11^ Notably, an increased incidence of BPD has been reported in this population,^12^^,^^13^ suggesting a potential link between disrupted bile acid metabolism and BPD pathogenesis. Concurrently, macrophages, whose recruitment and polarization (Macrophage1/inducible nitric oxide synthase [iNOS]+ vs. Macrophage2/cluster of differentiation 206 [CD206]+) are crucial for inflammatory responses^14^ and are known to be modulated by microbial metabolites, represent a potential mechanistic link.^15^ We therefore hypothesize that alterations in gut microbiota metabolites, including bile acids, contribute to BPD by dysregulating macrophage chemotaxis and polarization.

The present study aims to investigate the hypothesis that gut microbiota dysbiosis disrupts the balance of immunomodulatory microbiota metabolites, consequently influencing the inflammatory process in BPD by affecting macrophage function. Our objectives are to examine alterations in the gut microbiota and associated metabolites in infants with BPD and to explore the potential pathway linking gut microbiota-associated metabolites to lung inflammation in BPD through the modulation of macrophage function.

## Results

### Clinically relevant fecal microbiota composition

To explore BPD-associated bacteria, we collected stool samples and performed 16S ribosomal RNA (16S rRNA) sequencing in 30 infants with BPD and 33 control preterm infants. The perinatal and demographic attributes of the enrolled infants are summarized in Table 1. Infants with and without BPD did not differ significantly in sex, gestational age, birth weight, maternal characteristics, delivery mode, feeding mode, or comorbidity profiles (all *p* ≥ 0.05). However, infants with BPD exhibited significantly longer durations of mechanical ventilation and antibiotic exposure (*p* < 0.001 and *p* = 0.001, respectively).

**Table 1:** Demographic and clinical characteristics of the study infants (*N* = 63)

Parameters	BPD	Control	*p* value
	*n* = 30	*n* = 33	
Demographic data			
Birth weight (g)	1064.5 ± 214.3	1186.8 ± 287.5	0.059
Gestational age (weeks)	27.5 ± 2.0	28.7 ± 2.7	0.067
Sex			0.735
Male	16 (53.3%)	19 (57.6%)	
Female	14 (46.7%)	14 (42.4%)	
Mode of delivery			0.259
Vaginal	17 (56.7%)	14 (42.4%)	
Cesarean	13 (43.3%)	19 (49.2%)	
Maternal characteristics			
Age	32.7 ± 4.5	31.1 ± 5.1	0.182
Pregnant times	2 (1,3)	1 (1,2)	0.415
Labor times	1.5 (1,2)	1 (1,2)	0.416
*In vitro* fertilization	6 (20%)	4 (12.1%)	0.498
Prolonged rupture of membranes	2 (6.7%)	6 (18.2%)	0.261
Pregnant induced hypertension syndrome	6 (20%)	4 (12.1%)	0.498
Gestational diabetes mellitus	4 (13.3%)	5 (15.2%)	1.000
Maternal antibiotic use prior to birth	3 (10.0%)	4 (12.1%)	1.000
Antenatal corticosteroids	13 (43.3%)	10 (30.3%)	0.283
Clinical presentations			
Apgar score at 5min (≤7)	16 (53.3%)	15 (45.5%)	0.532
Feeding			0.071
Breast milk	14 (46.7%)	7 (21.2%)	
Mixed	4 (13.3%)	4 (66.7%)	
Formula milk	12 (40.0%)	22 (12.1%)	
Duration of mechanical ventilation (days)	17.0 (6.8, 30.8)	7.0 (2.0, 12.0)	***<0.001***
Duration of CPAP (days)	16.0 (8.0, 23.0)	8.0 (6.0, 11.5)	***0.038***
Days of antibiotic use	36.5 (27.8, 48.0)	25.0 (19.5, 34.0)	***0.001***
Neonatal respiratory distress syndrome	15 (50.0%)	12 (36.4%)	0.275
Patent ductus arteriosus	13 (43.3%)	11 (33.3%)	0.414
Necrotizing Enterocolitis	5 (15.7%)	5 (15.2%)	0.869
Sepsis	9 (30.0%)	6 (18.2%)	0.271

Values in bold-italic indicate statistical significance (*p* < 0.05).

Data are presented as mean ± SD, N (%), or median (IQR).

BPD, bronchopulmonary dysplasia; CPAP, continuous positive airway pressure.

**Figure 1:**
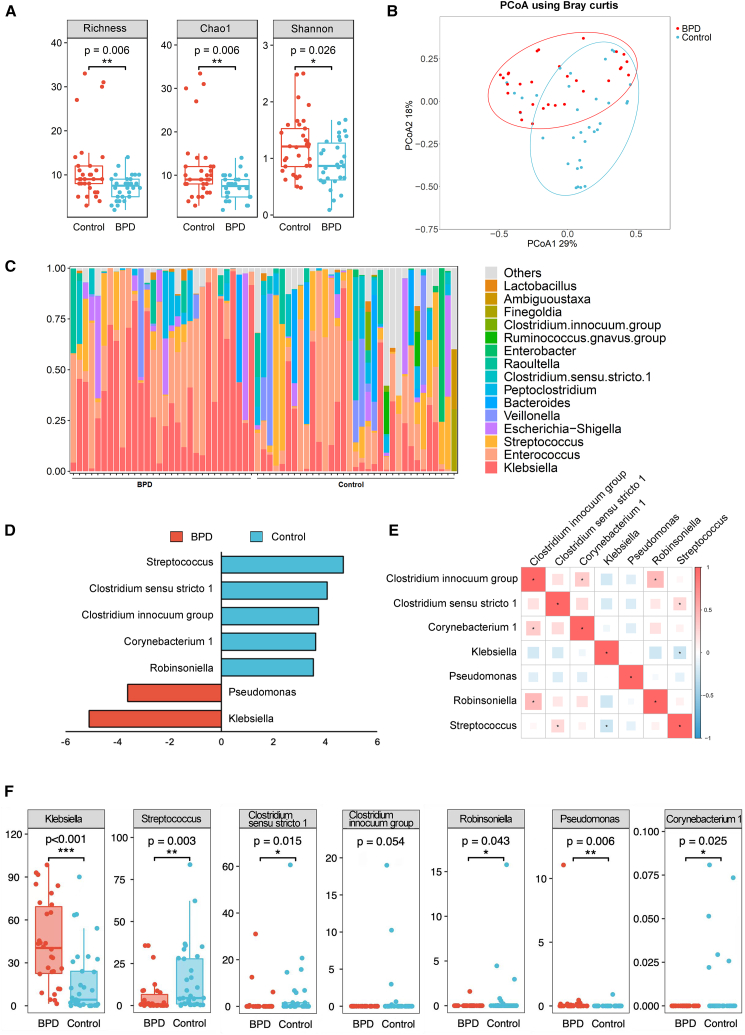
Alterations in intestinal microbiota in patients with BPD (A) Alpha diversity comparison between patients with BPD and preterm controls measured by OTU richness, Chao1, and Shannon indexes (Mann-Whitney test; *p* = 0.006, 0.006, and 0.026; FDR-adjusted *p* = 0.03, 0.03, 0.043, respectively). (B) Beta diversity comparison generated using PCoA of Bray-Curtis distances, with taxa summarized at the genus level. Each point corresponds to a sample, shaped and colored by group. (C) Stacked bar chart showing the relative abundances of the top 15 bacterial genera. (D) LEfSe-generated linear discriminant analysis effect size plot comparing the microbiome between the two groups. (E) Correlation among bacterial taxa with significant differences using Spearman’s correlation method. Red and blue represent positive and negative correlations, respectively. (F) Significant differences in gut bacterial taxa between groups. Specific FDR-adjusted *p*-values: *Klebsiella* (<0.001), *Streptococcus* (0.011), *Clostridium sensu stricto 1* and *Corynebacterium 1* (both 0.026), *Clostridium innocuum group* (0.033), *Robinsoniella* (0.036), and Pseudomonas (0.014). Comparisons were performed using the Mann-Whitney test. ∗, *p* < 0.05; ∗∗, *p* < 0.01; ∗∗∗, *p* < 0.001.

There was a significantly decrease in alpha diversity, as indicated by richness (*p* = 0.006 and false discovery rate [FDR]-adjusted *p* = 0.03), Chao (*p* = 0.006 and FDR-adjusted *p* = 0.03), and the Shannon index (*p* = 0.026 and FDR-adjusted *p* = 0.043), in patients with BPD compared with preterm controls (Figure 1A). In addition, principal coordinates analysis (PCoA) illustrated a distinct segregation in gut microbiota composition between the BPD and preterm control groups (Figure 1B). The relative abundance of different bacterial genera in both groups is shown in Figure 1C.

At the genus level, seven bacterial taxa exhibited notable differences in enrichment discrepancies between patients with BPD and controls. *Streptococcus*, *Clostridium sensu stricto* 1, *Clostridium innocuum group*, *Corynebacerium* 1, and *Robinsoniella* were significantly decreased, whereas *Pseudomonas* and *Klebsiella* were elevated in patients with BPD (all *p* < 0.05 and FDR-adjusted *p* < 0.05, Figure 1D). The correlations among specific bacterial species are shown in Figure 1E. More detailed information on the relative abundance of each genus by case-control status is presented in Figure 1F. Notably, *Klebsiella* and *Streptococcus* exhibited higher abundance levels than the other five genera.

### Fecal microbiota-derived metabolites in patients with BPD

**Figure 2:**
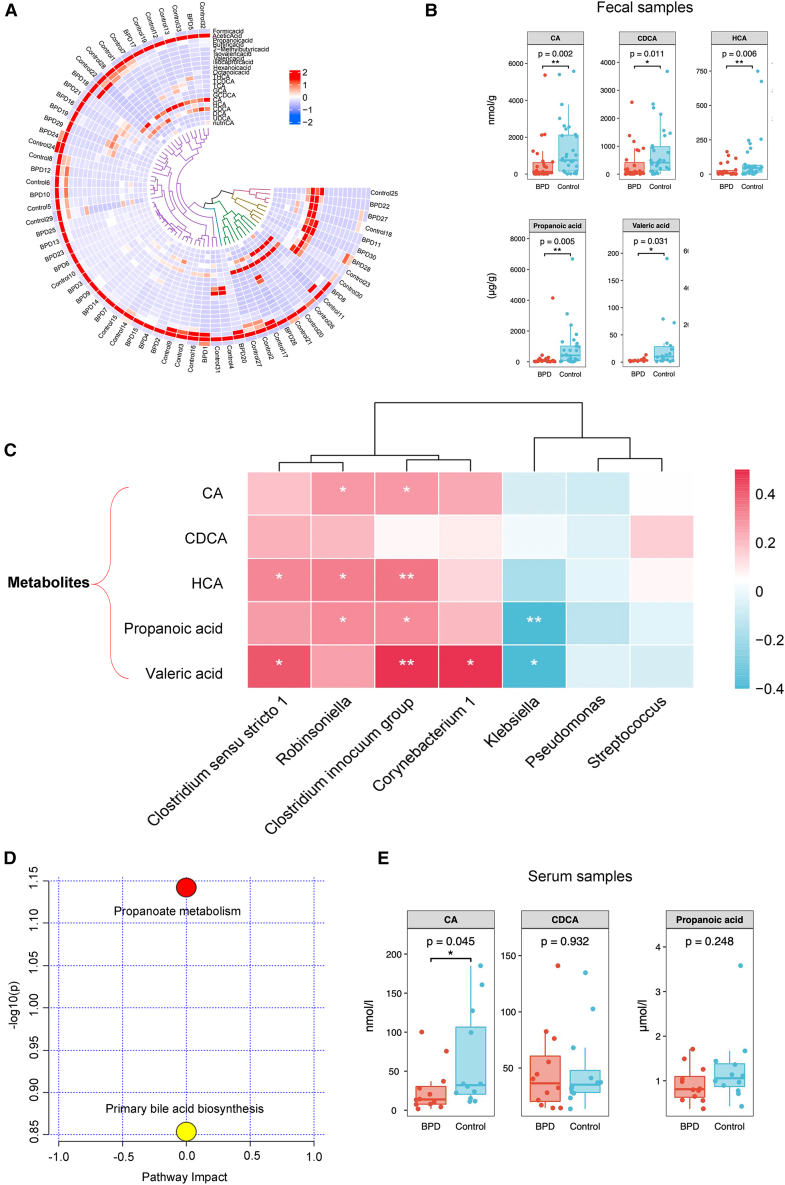
Altered microbial metabolic profiles in gut and serum (A–D) Analysis of metabolites in fecal samples from patients with BPD (*n* = 30) and controls (*n* = 33). (A) Circular heatmap representing the relative abundance of metabolites, including short-chain fatty acids and bile acids, in the fecal samples. (B) Key gut metabolites with significant intergroup differences. FDR-adjusted *p* values: CA (0.015), CDCA (0.015), HCA (0.015), propanoic acid (0.015), and valeric acid (0.031). (C) Spearman correlation analysis between seven bacterial taxa and five metabolites with significant differences between the two groups. Red and blue represent positive and negative correlations, respectively. (D) KEGG pathway analysis of the five significantly different fecal metabolites. Two pathways were detected as significant. (E) Quantification of three major metabolites in the serum of 12 patients with BPD and 12 controls. Only CA showed a significant difference between the two groups. (Mann-Whitney test) (*p* = 0.045). *∗p* < 0.05; ∗∗, *p* < 0.01.

According to previous studies, these differential microbiotas, such as *Klebsiella* and *Clostridium* species, are associated with the synthesis of BAs and SCFAs.^16^^,^^17^^,^^18^ Thus, we further investigated these metabolites in feces and explored their associations with BPD. We initially screened 15 SCFA and 32 BA standard compounds via targeted metabolomics, among which a total of 10 SCFAs and 11 BAs were successfully identified and quantified (Table S1). The distribution of metabolites in each fecal sample is shown in Figure 2A. Comparing individuals with BPD and control individuals, we observed a significant decrease in the levels of cholic acid (CA) (*p* = 0.002 and FDR-adjusted *p* = 0.15), chenodeoxycholic acid (CDCA) (*p* = 0.011 and FDR-adjusted *p* = 0.015), hyodeoxycholic acid (HCA) (*p* = 0.006 and FDR-adjusted *p* = 0.15), propanoic acid (*p* = 0.005 and FDR-adjusted *p* = 0.015), and valeric acid (*p* = 0.031 and FDR-adjusted *p* = 0.031) (Figure 2B and Table S2). After adjustment for key clinical covariates, including gestational age, birth weight, sex, feeding mode, ventilation/continuous positive airway pressure (CPAP) duration, and antibiotic days, these metabolites remained significantly different (Table S3).

Spearman’s correlation analyses at the genus level suggested positive correlations of *Robinsoniella* and the *Clostridium innocuum* group with CA, HCA, and propanoic acid. Conversely, negative correlations were observed between *Klebsiella* and propanoic acid, as well as valeric acid (Figure 2C). Kyoto Encyclopedia of Genes and Genomes (KEGG) analysis of fecal metabolomics data was conducted to identify biologically relevant pathways and guide key metabolite selection for targeted blood quantification, uncovering two major BPD-related pathogenic pathways: propanoate metabolism and primary bile acid biosynthesis (Figure 2D). Propanoic acid, CA, and CDCA were confirmed as key metabolites in these pathways.

To translate these fecal metabolite findings to their systemic relevance, we quantified these metabolites in serum. The representativeness of the serum subcohort and the comparability of the BPD and control groups were first verified (Tables S4 and S5). Subsequent analysis confirmed significantly lower circulating CA levels in the BPD group (Figure 2E and Table S6). This systemic deficiency, coupled with the prior microbiome and metabolome data, supports the hypothesis that impaired microbial production of CA is involved in the development of BPD.

### CA attenuates lung inflammation

**Figure 3:**
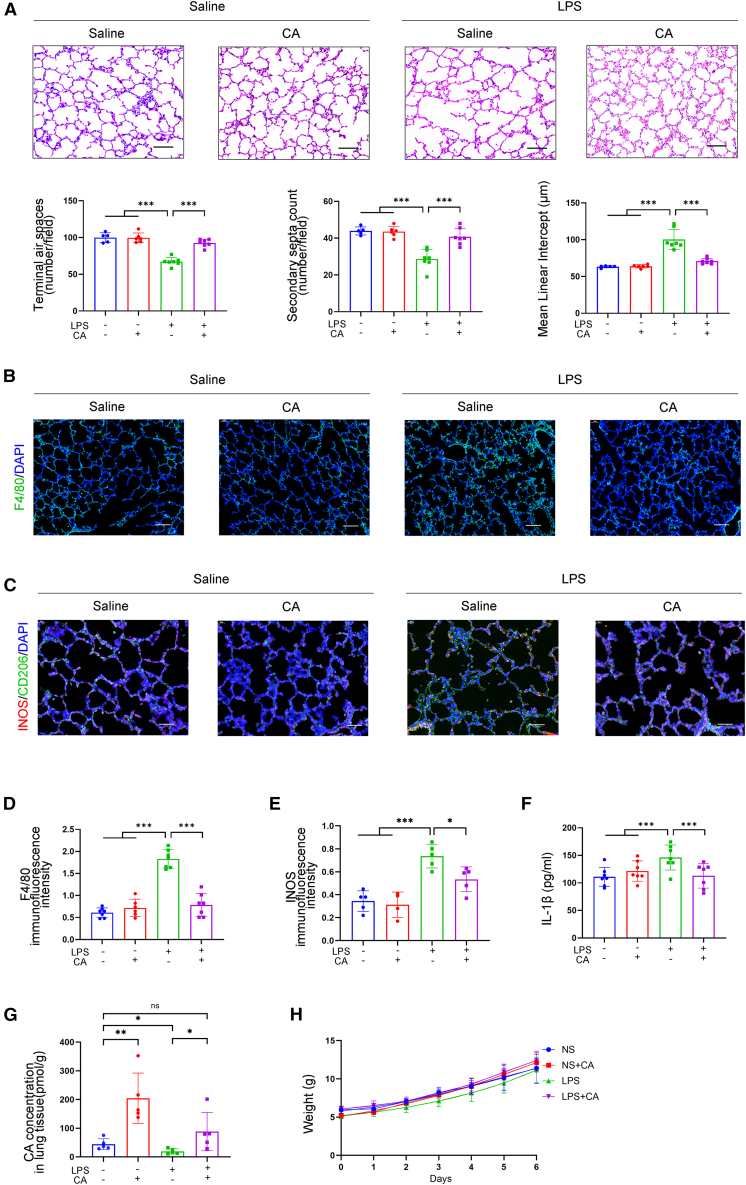
Cholic acid feeding protected against lung inflammation Microscopy of the left lung from BPD rats induced by chorioamnionitis or saline controls, with feeding of CA or saline for 7 days. (A) Representative lung sections stained with H&E. Scale bars, 50 μm. Quantification of terminal airspace, secondary septa, and mean linear intercept of lung tissues from P6 (*n* = 5–7). (B and C) Representative images of lung tissues immunostained for F4/80 and iNOS. Scale bars, 50 μm. (D and E) Quantitation of F4/80 and iNOS fluorescence intensity (*n* = 5–7). (F) IL-1β protein levels in rat lung tissues (*n* = 5–7). (G) Quantitative analysis of CA concentrations in rat lung tissues (*n* = 3). (H) Comparable growth curves across all experimental group (*n* = 5). All data are expressed as mean ± SD ∗*p* < 0.05; ∗∗, *p* < 0.01; ∗∗∗, *p* < 0.001.

To elucidate the regulatory effect of CA on BPD progression, we carried out histological evaluations of lung tissues from chorioamnionitis-induced BPD rats with or without oral CA supplementation. Lung tissue samples from the BPD rat model revealed alveolar simplification, including enlarged alveoli, decreased terminal airspaces, and reduced secondary septa. In contrast, these features were dampened in the CA-treated BPD groups (Figure 3A).

Next, we investigated whether CA modulates macrophage accumulation in the lung. Immunofluorescence analyses targeting F4/80^+^ (total macrophages) and iNOS^+^ (pro-inflammatory macrophages) revealed that lipopolysaccharide (LPS) exposure triggered a marked increase in pulmonary macrophage accumulation, with a prominent elevation in the iNOS^+^ pro-inflammatory subset (Figures 3B and 3C). This increase was substantially reversed by CA treatment (Figures 3D and 3E). Given the established role of iNOS^+^ macrophage-derived interleukin-1 beta (IL-1β) in driving BPD development,^19^^,^^20^ we quantified IL-1β levels and found a significant decrease in the CA-treated BPD group (Figure 3F). To rule out neutrophil-mediated effects, supplementary immunofluorescence analyses were performed to quantify pulmonary neutrophil populations; no significant differences in neutrophil counts were observed between the BPD model and CA-treated BPD groups (Figure S1).

We also quantified CA concentrations in lung tissues to confirm target organ delivery. Rats in the BPD model exhibited significantly reduced lung CA levels, while oral CA administration effectively elevated lung CA concentrations to levels comparable with control rats, with no significant difference between CA-treated BPD and control groups post-supplementation (Figure 3G). Additionally, growth curves were comparable across all four experimental groups, ruling out potential side effects of CA (Figure 3H). Collectively, these findings support that CA exerts potent anti-inflammatory effects during BPD progression, predominantly mediated through the modulation of pulmonary macrophage infiltration and pro-inflammatory activation.

### CA reduced macrophage accumulation in the lung in the BPD model through CCR5 signaling

**Figure 4:**
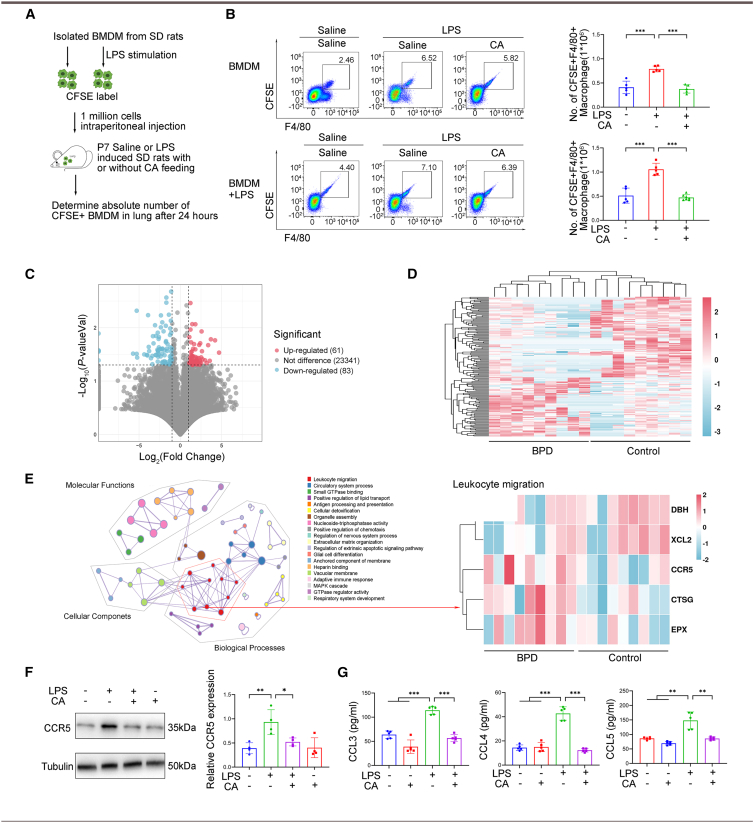
Cholic acid reduced macrophage accumulation in the lung via CCR5 signaling in a rat BPD model induced by chorioamnionitis (A) Diagram showing the generation and labeling of bone marrow-derived macrophages migrate to the lungs of BPD rats induced by chorioamnionitis, with or without CA feeding, and controls. See Macrophage migration test in the STAR Methods section. (B) Quantitative analysis of the number of CFSE-stained F4/80 macrophages in rat lungs (*n* = 5–7 rats/group). (C–E) RNA sequencing of whole peripheral blood from 9 patients with BPD and 9 controls. (C) Volcano plot displaying differentially abundant genes between the two groups. Each dot represents a gene, with red dots for genes significantly upregulated and blue dots for genes significantly downregulated in infants with BPD. (D) Representative heatmaps for genes with significant differences between the two groups. (E) Gene interaction map of functional modules. Red plots represent genes involved in leukocyte migration, among which five genes were significantly up-and downregulated, including CCR5. (F and G) Experiments on CCR5 signaling in the lungs of BPD rats induced by chorioamnionitis or saline controls, with or without CA feeding for 7 days. (F) Representative immunoblot and relative CCR5 expression in whole lung lysates of P6 rats (*n* = 4). (G) Levels of CCL3, CCL4, and CCL5 in rat lung tissues (*n* = 5). Data are expressed as mean ± SD. ∗*p* < 0.05; ∗∗, *p* < 0.01; ∗∗∗, *p* < 0.001.

During inflammation, recruited monocytes play a crucial role in establishing the lung macrophage pool.^21^ To investigate the mechanisms underlying the effects of CA on macrophage migration, we conducted a macrophage migration assay (Figure 4A) and observed that both bone marrow-derived macrophages (BMDMs) and LPS-stimulated BMDMs from CA-treated newborn BPD rats exhibited a decreased ability to traffic to the lungs compared with BPD rats induced by chorioamnionitis (Figure 4B). Only a small number of labeled macrophages migrated to the small intestine and spleen (Figure S2). These results suggest that CA treatment reduces macrophage accumulation in the lungs in the context of BPD.

Subsequently, to investigate migration-related mRNAs in inflammatory cells associated with BPD, we performed whole blood RNA sequencing in 9 BPD cases and 9 control infants matched for gestational age, sex, and birth weight (*p* > 0.05, Table S7). The volcano plot displayed significant differential expression of 144 mRNAs, including 61 upregulated and 83 downregulated mRNAs in patients with BPD compared with preterm controls (Figure 4C), and cluster analysis with a heatmap illustrated the differentially expressed genes (Figure 4D). Subsequent Gene Ontology (GO) analysis focused on selected terms with the most significant *p* values from each of the 20 clusters. Within the cluster associated with leukocyte migration, we detected five genes exhibiting differential expression in patients with BPD. Specifically, DBH and XCL2 were found to be downregulated, while CCR5, CTSG, and EPX were upregulated (Figure 4E). In conjunction with our previous experiments, these findings suggest that C-C chemokine receptor type 5 (CCR5) signaling may promote macrophage accumulation and contribute to alveolar developmental arrest.^22^

Prompted by these findings, we assessed the CCR5 axis *in vivo*. CA supplementation significantly reduced lung CCR5 protein expression (Figure 4F) and the levels of its ligands CCL3, CCL4, and CCL5 (Figure 4G) in BPD rats. Collectively, these results suggest that CA curtails macrophage accumulation in the lung, at least in part, by suppressing the CCR5 signaling pathway.

### CA suppressed CCR5 expression and iNOS+ phenotype of macrophages via HIF-1 alpha signaling

**Figure 5:**
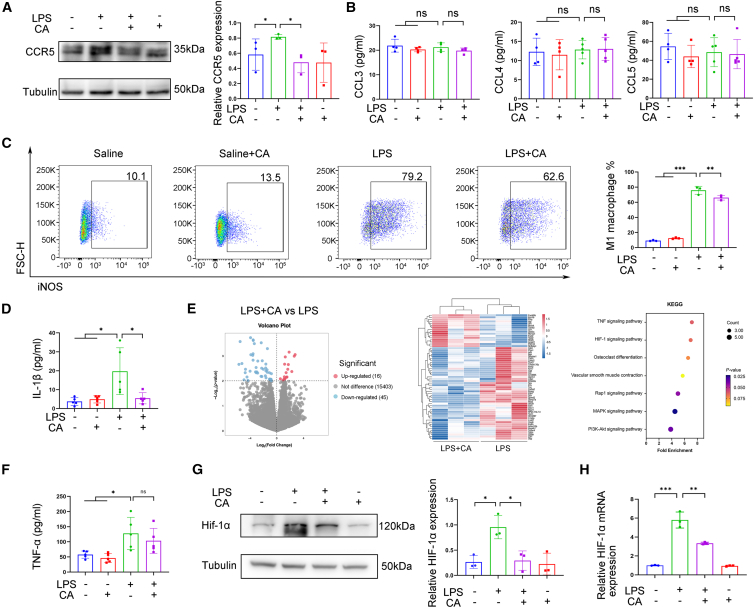
Cholic acid could regulates macrophage plasticity and function (A–D) RAW 264.7 macrophage cell lines were cultured in the presence or absence of CA for 2 h and stimulated with or without LPS. (A) Representative immunoblot and relatively CCR5 expression analysis (*n* = 3). (B) Analysis of CCL3, CCL4, and CCL5 levels in culture supernatant (*n* = 4). (C) Representative flow cytometry plot of iNOS^+^ macrophages and quantitative analysis among each group (*n* = 3). (D) Analysis of IL-1β level in suspension. (*n* = 5). (E) RNA sequencing of cells isolated from bronchoalveolar lavage fluid (BALF) from the lungs of BPD rats induced by chorioamnionitis, with or without CA feeding (*n* = 3 rats/group). Volcano plot displays differentially abundant genes between LPS and LPS + CA feeding groups. Representative heatmaps of genes with significant differences between the two groups are shown. KEGG pathway analysis showed TNF and HIF-1 pathways as significantly involved. (F and G) RAW 264.7 cells cultured in the presence or absence of CA for 2 h and stimulated with or without LPS. (F) Analysis of TNF-α levels in suspension (*n* = 5). (G) Representative immunoblot and analysis of relatively HIF-1α expression (*n* = 3). (H) Relative HIF-1α mRNA expression measured by RT-qPCR (*n* = 3). All data are expressed as mean ± SD. ∗*p* < 0.05; ∗∗, *p* < 0.01; ∗∗∗, *p* < 0.001.

Further *in vitro* experiments in RAW 264.7 cells confirmed that CA intervention significantly attenuated the LPS-induced upregulation of CCR5 expression (Figure 5A). Notably, CA did not reduce the LPS-stimulated expression of the CCR5 ligands C-C motif chemokine ligand 3 (CCL3), C-C motif chemokine ligand 4 (CCL4), and C-C motif chemokine ligand 5 (CCL5) (Figure 5B), suggesting a specific regulatory effect on the receptor itself. Concurrently, CA treatment markedly reduced the proportion of iNOS^+^ macrophages and the secretion of the key pro-inflammatory cytokine IL-1β (Figures 5C and 5D). These findings indicate that CA significantly modulates macrophage chemotaxis, polarization, and inflammatory activity.

To investigate potential upstream regulators mediating CA’s effects, bronchoalveolar lavage fluid (BALF) was isolated from BPD model pups with or without CA treatment. BALF samples were centrifuged to pellet cells, and total RNA was extracted from the cell pellet using TRIzol reagent for RNA sequencing. Volcano plot analysis revealed 61 differentially expressed genes between the two groups. KEGG pathway enrichment analysis suggested that these differentially expressed genes were predominantly associated with the tumor necrosis factor (TNF) and hypoxia-inducible factor 1 (HIF-1) signaling pathways (Figure 5E). Subsequent validation showed that, while CA did not alter LPS-elevated TNF-α levels (Figure 5F), it effectively suppressed the LPS-induced increase in both HIF-1α protein and mRNA expression in RAW 264.7 cells (Figures 5G and 5H). As BAs typically signal via the nuclear receptors farnesoid X receptor (FXR) or Takeda G protein-coupled receptor 5 (TGR5), we assessed their involvement. Western blot analysis confirmed the presence of both receptors in neonatal rat lung tissue, but their expression levels showed no significant differences between experimental groups (Figure S3).

**Figure 6:**
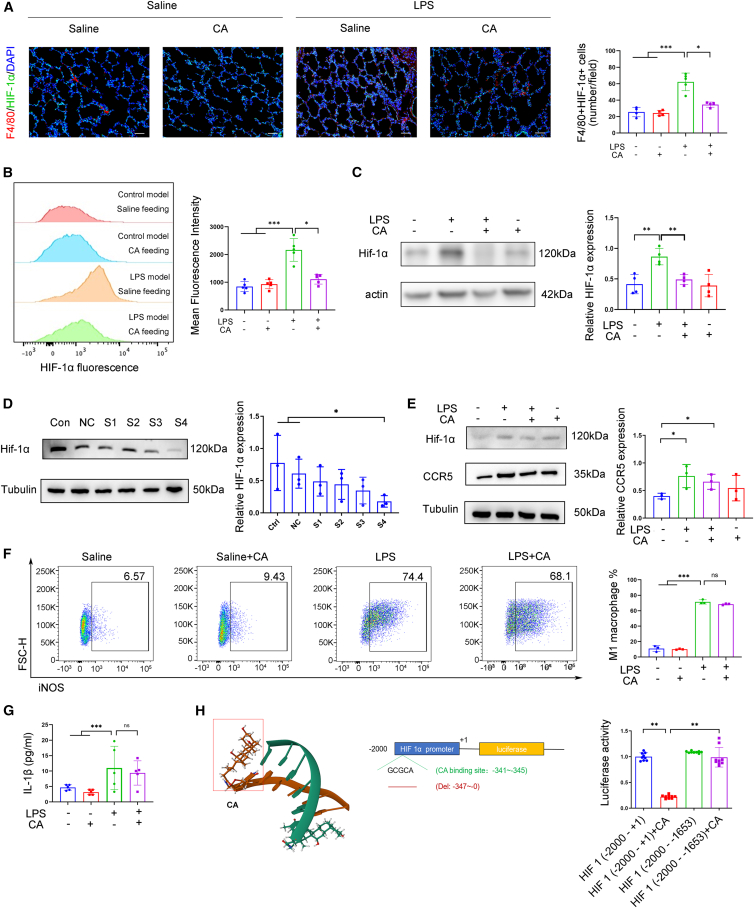
Cholic acid modulates macrophage function via interaction with HIF-1α (A) Representative images of lung tissues immunostained for F4/80 and HIF-1α in BPD rats induced by chorioamnionitis or saline controls, with feeding of CA or saline for 7 days. Quantitation of F4/80+ HIF-1α+ cell numbers (*n* = 4–5). Scale bars, 20 μm. (B) HIF-1α fluorescence intensity in F4/80+cells isolated from BALF of BPD rats induced by chorioamnionitis, with CA or saline feeding for 7 days (*n* = 5). (C) Representative immunoblot and relative HIF-1α expression analysis in bone marrow-derived macrophages cultured with or without CA for 2 h and stimulated with or without LPS (*n* = 4). (D) HIF-1α was stably knocked down in RAW 264.7 cells using siRNA transfection. Representative immunoblot and relatively HIF-1α expression analysis show that HIF-1α-siRNA(S4) had the highest knockdown efficiency (*n* = 3). (E–G) HIF-1α knockdown RAW 264.7 cells were cultured with or without CA for 2 h and stimulated with or without LPS (*n* = 3). Representative immunoblot and relative CCR5 expression analysis (*n* = 3) (E) Representative flow cytometry plot of iNOS^+^ macrophages and quantitative analysis (*n* = 3). (F) Analysis of IL-1β levels (*n* = 5) (G). All analyses were performed using HIF-1α knockdown cells. (H) Transcription activity of CA on the HIF-1α promoter, as determined by a luciferase reporter assay. Left: schematic depicting the predicted CA-binding site on HIF-1α. Middle: plasmid encoding luciferase containing the DNA fragment without the predicted binding site (−341 to −345). Right: relative luciferase activities of the deletion constructs as determined by luciferase reporter assay (*n* = 9). All data are expressed as mean ± SD. ∗*p* < 0.05; ∗∗, *p* < 0.01; ∗∗∗, *p* < 0.001.

Next, we evaluated the impact of CA on HIF-1 modulation. Immunofluorescence analysis of lung tissue revealed that LPS exposure significantly increased the number of F4/80^+^HIF-1α^+^ macrophages, an effect that was potently reversed by CA treatment (Figure 6A). Immunofluorescence analysis of BALF-isolated cells further demonstrated a marked elevation in HIF-1α fluorescence intensity within macrophages of the LPS-stimulated group, which was effectively mitigated by CA administration (Figure 6B). Consistently, *in vitro* experiments showed that CA treatment of LPS-stimulated BMDMs significantly suppressed the LPS-induced upregulation of HIF-1α, aligning with *in vivo* observations (Figure 6C), suggesting that CA is associated with reduced HIF-1α expression.

Additionally, we designed four types of HIF-1α small interfering RNA (siRNA) and transferred them into RAW264.7 cells, finding that HIF-1α-siRNA (S4) had the highest knockdown efficiency (Figure 6D and Table S8). Crucially, in HIF-1α-silenced cells, CA lost its ability to inhibit LPS-induced CCR5 upregulation, iNOS^+^ macrophage polarization, and IL-1β production (Figures 6E–6G). These findings imply that the suppressive effects of CA on macrophage chemotaxis and inflammation are mediated through HIF-1α downregulation.

Based on these results, we hypothesized that CA might regulate HIF-1α transcription. Bioinformatic analysis of the HIF-1α promoter identified a putative CA-binding site (GCGCA) at positions −341 to −345 (Figure 6H). Luciferase reporter assays revealed that deletion of this specific site abolished the CA-mediated suppression of promoter activity, whereas the full-length promoter remained responsive (Figure 6H). These results suggest that this *cis*-regulatory region is essential for CA-mediated suppression of HIF-1α transcriptional activation.

## Discussion

In our study, we observed significant differences in the gut microbiota and bacterial communities between infants with BPD and preterm control groups. Through comprehensive analysis of the metabolome and gut microbiota in infants with BPD, we observed specific gut microbiota-associated metabolites that were significantly reduced in BPD. These findings provide new insights into the mechanistic links between gut microbiota, microbiota-derived metabolites, and pulmonary inflammation in the development of BPD. We found that specific gut microbiota-related metabolites, namely CA, could improve BPD by reducing CCR5 expression, IL-1β production, and M1(iNOS+) macrophage polarization in LPS-exposed macrophages via the HIF-1 signaling pathway. To our knowledge, this is the first study to analyze the gut microbiota-metabolite association network in fecal samples from patients with BPD. Our results suggest that the gut microbiota is associated with BPD, with a potential correlational link involving CA.

Our findings reinforce the association between gut dysbiosis and BPD. While gut microbial alterations in preterm infants with BPD have been reported,^23^ our study delineates specific taxonomic shifts. A key finding is the elevated abundance of *Klebsiella*, a genus previously noted in similar contexts.^24^ This Gram-negative bacterium is a recognized pathogen,^25^ and its LPS component is a potent inducer of the inflammatory responses implicated in BPD.^26^ In parallel, we found a depletion of *Streptococcus*, a core genus in the healthy infant gut.^27^ The negative correlation between *Streptococcus* and *Klebsiella*, along with analogous findings in necrotizing enterocolitis models,^28^ points to disruption of normal intestinal homeostasis. However, our analysis did not reveal differences in other sepsis-related pathogens*,* such as *E. coli* or group B *Streptococcus*, which have been tied to BPD risk.^29^ This discrepancy may be attributed to the modest scale and single-center nature of our study, highlighting the need for future investigation with expanded cohorts.

Moreover, in contrast to a prior report that found no significant difference in SCFAs at day 14,^23^ our profiling revealed significantly lower fecal levels of key SCFAs (propanoic acid, valeric acid) and primary BAs (CA, CDCA, HCA) in infants with BPD. These metabolites were linked to specific bacterial shifts. For example, the *Clostridium innocuum group* (within *Clostridiales*) positively correlated with CA and propanoic acid, aligning with the known role of *Clostridiales* in propionate production.^18^ Importantly, *Clostridium* can contribute to the deconjugation of conjugated BAs by removing glycine and taurine, reverting them into primary BAs.^30^^,^^31^ Thus, depletion of *Clostridium* may be associated with reduced production of primary BAs, such as CA. Given that intestinal BAs enter the portal circulation to exert systemic effects,^32^^,^^33^ we assessed serum levels and confirmed a parallel decrease in CA in infants with BPD. This congruence between fecal and serum metabolomics data underscores the translational relevance of CA, leading us to select its metabolic pathway for in-depth study.

Subsequent investigations confirmed that CA supplementation effectively ameliorated lung pathology in LPS-challenged rats. Notably, while lung CA levels were significantly reduced in the BPD model, they were restored to control levels upon supplementation, providing direct evidence of CA’s effective delivery and accumulation in the target organ. Inflammation is a core driver of BPD, with gut microbiota-derived metabolites increasingly recognized as key modulators of macrophage-mediated immunity.^34^ This aligns with reported cytokine dysregulation, such as elevated IL-1β, TNF-α, in BPD,^35^ where pathogenic macrophage polarization exacerbates inflammatory injury and disrupts lung maturation.^36^^,^^37^ Thus, targeting macrophages represents a rational therapeutic strategy.^19^ In the chorioamnionitis-induced BPD model, the increase in pulmonary macrophage numbers is probably attributed to the recruitment of bone marrow-derived monocytes/macrophages. In our study, CA treatment significantly reduced pulmonary macrophage accumulation and IL-1β levels, indicating its ability to mitigate inflammation and promote alveolarization. Intriguingly, whole-blood RNA sequencing in patients with BPD revealed marked upregulation of CCR5, a chemokine receptor critical for monocyte/macrophage recruitment and inflammation,^38^^,^^39^ corroborating our prior findings.^22^ *In vitro*, CA downregulated LPS-induced CCR5 expression in macrophages and suppressed M1 (iNOS^+^) polarization and IL-1β release. These coordinated effects pointed to a common upstream regulator. RNA sequencing of BALF cells subsequently identified the HIF-1 signaling pathway as a critical upstream node mediating CA’s regulation of macrophage function in BPD.

HIF-1α plays a dual role in vascular homeostasis and immune regulation. While it is essential for physiological angiogenesis, its persistent or pathological overexpression is implicated in conditions such as pulmonary hypertension,^40^ a vascular abnormality often associated with BPD. In this study, we focused on its pathological activation in inflammatory macrophages during lung injury, distinct from its physiological developmental functions. Our data demonstrate that CA effectively attenuates LPS-induced HIF-1α overexpression without affecting baseline levels, thereby preserving its essential functions. This context-dependent suppression is critical, as HIF-1α drives pro-inflammatory macrophage polarization and cytokine production, such as IL-1β.^41^^,^^42^ Thus, our intervention selectively targets pathological HIF-1α overactivation. Mechanistically, findings from HIF-1α-siRNA and luciferase assays suggest that CA may regulate HIF-1α-mediated transcriptional activity, which likely contributes to the observed downregulation of HIF-1α. This effect of CA could, in turn, influence macrophage chemotaxis and M1 (iNOS^+^) polarization, potentially attenuating inflammatory responses and supporting the restoration of immune homeostasis.

In conclusion, our study demonstrates that infants with BPD exhibit significant dysbiosis, characterized by distinct gut microbial communities and associated metabolites. Importantly, we establish a connection between the gut microbiota, its metabolites, and macrophage function. These findings provide valuable clues for potential therapeutic approaches targeting macrophage metabolism to address the inflammatory challenges in BPD. By understanding the intricate interplay between the gut microbiota, metabolites, and immune function, novel interventions may be developed to mitigate the impact of BPD and improve patient outcomes.

### Limitations of the study

Several limitations of this study should be acknowledged. First, the relatively small cohort of patients with BPD may introduce potential selection bias, which requires further validation in larger, multicenter populations. Second, due to technical challenges in genetically manipulating primary macrophages, we used immortalized macrophage cell lines for preliminary mechanistic exploration. Nevertheless, subsequent *in vivo* functional assays and primary cell verification supplemented this limitation and confirmed CA-mediated regulation of HIF-1α. Third, our study design did not fully capture the dynamic fluctuations of the gut microbiota; however, the rodent model retains key pathophysiological features of human BPD and supports the therapeutic effect of CA. Fourth, owing to the extremely early developmental stage of the neonatal pups, we were unable to reliably determine their sex. Consequently, we could not evaluate sex-specific responses to CA intervention or explore whether the therapeutic effects differed between male and female offspring. Further studies incorporating sex-stratified analyses are therefore warranted to validate and expand our findings. Finally, inconsistencies in sampling time points and experimental contexts between human and murine data limit direct cross-species comparison and translational application. Future studies should standardize sampling schedules and unify experimental settings to address these issues.

## Resource availability

### Lead contact

Further information and requests for resources and reagents should be directed to and will be fulfilled by the lead contact, Yongjun Zhang (zhangyongjun@sjtu.edu.cn).

### Materials availability

This study did not generate new unique reagents.

### Data and code availability

• Human whole blood RNA-seq and BALF bulk RNA-seq data have been deposited in the NCBI Sequence Read Archive (SRA): PRJNA1394681 and PRJNA1394643, respectively.
• Fecal 16S rRNA gene sequencing data and metabolomics raw data are publicly available via Mendeley Data (https://doi.org/10.17632/z8skphy8xw.1).
• Any additional information required to reanalyze the data reported in this paper is available from the lead contact upon request.

## Acknowledgments

This work was supported in part by grants from the 10.13039/501100001809National Natural Science Foundation of China (81800001 to D. Zhao, 82271745 to Y.Z., and 82371721 to X.W.).

The authors thank Zhongcheng Luo (Lunenfeld-Tanenbaum Research Institute, Mount Sinai Hospital, 10.13039/501100003579University of Toronto, Toronto, Canada) for assistance with English language editing.

## Author contributions

Y.Z. and X.W. designed the study. L.S., L.C., D. Zhao, C.G., X.Z., and C.L. performed the experiments. C.G. and D. Zhao assisted with patient sample experiments. D. Zhao, X.Z., and C.L. contributed to the animals and cell experiments. L.C., C.G., and D. Zhu assisted with sequencing analysis. C.G., D. Zhu, J.S., and L.S. analyzed the data. D. Zhao and X.W. wrote the manuscript. Y.Z. oversaw the project.

## Declaration of interests

The authors declare no competing interests.

## STAR★Methods

### Key resources table

**Table:** 

REAGENT or RESOURCE	SOURCE	IDENTIFIER
**Antibodies**		

F4/80 antibody	Santa Cruz Biotechnology	Cat # sc-377009; RRID: AB_2927461, Cat # sc-52664; RRID: AB_629466
Anti-iNOS antibody	Abcam	Cat # ab178945; RRID: AB_2861417
PE anti-Nos2 (iNOS) Antibody	Biolegend	Cat # 696806; RRID: AB_2876745
FITC anti-CD206 (MMR) Antibody	Biolegend	Cat # 141703; RRID: AB_10900988
CCR5 Antibody	Affinity Bioscience	Cat # AF6339; RRID: AB_2835195
Alpha Tubulin antibody	Proteintech	Cat # 66031-1-Ig; RRID: AB_11042766
HIF-1 alpha Antibody	Cell Signaling Technology	Cat # 36169; RRID: AB_2799095
TGR5 antibody	Novus Biologicals	Cat # NBP2-23669; RRID: AB_3073863
FXR antibody	Proteintech	Cat # 25055-1-AP; RRID: AB_2879874

**Chemicals, peptides, and recombinant proteins**		

Lipopolysaccharide (LPS, E. coli 055:B5)	Sigma-Aldrich	L2880
Cholic acid (CA)	Sigma-Aldrich	C1129
TRIzol reagent	Sigma-Aldrich	T3934
Type VIII Collagenase	Sigma-Aldrich	C2139
DNase I	Sigma-Aldrich	DN25
Percoll layered liquid	Sigma-Aldrich	GE17-0891-01
Halt™ Protease Inhibitor Cocktail (100×)	Thermo Scientific	87786
Lipofectamine 3000	Invitrogen	L3000015

**Critical commercial assays**		

QIAamp PowerFecal DNA Kit	Qiagen	12830–50
CellTrace™ CFSE Cell Proliferation Kit	Invitrogen	c34554
ELISA kits IL-1β(rat/mouse)	R&D Systems/ELK Biotechnology	SRLB00/ELK1118
ELISA kits CCL3(rat/mouse)	MultiSciences/ELK Biotechnology	70-EK361-96/ELK1116
ELISA kits CCL4(rat/mouse)	Bosterbio/ELK Biotechnology	EK0939/ELK1118
ELISA kits CCL5(rat/mouse)	MultiSciences/ELK Biotechnology	70-EK3129-96/ELK10193
ELISA kits TNF-α	ELK Biotechnology	ELK1387

**Experimental models: organisms/strains**		

Sprague–Dawley rats	Shanghai Laboratory Animal Center	SD strain

**Experimental models: cell lines**		

Human: HEK293T cells	ATCC	CRL-3216
Mouse: RAW264.7 cells	ATCC	TIB-71

**Oligonucleotides**		

16S rRNA V1–V3: 27F/534R	Custom synthesized	27F: 5′-AGAGTTTGATCCTGGCTCAG-3′ 534R: 5′-ATTACCGCGGCTGCTGG-3′
HIF-1α qPCR primers	Custom synthesized	F: GTATTATTCAGCACGACTT; R: GAGACATTGCCAGGTTTAT

**Deposited data**		

Human whole blood RNA-seq	NCBI Sequence Read Archive	PRJNA1394681
BALF bulk RNA-seq	NCBI Sequence Read Archive	PRJNA1394643
Fecal 16S rRNA gene sequencing and metabolomics	Mendeley Data	https://doi.org/10.17632/z8skphy8xw.1

**Software and algorithms**		

IBM SPSS software, version 20.0	SPSS, Inc., Chicago, IL, USA	https://www.ibm.com/spss
R version 4.0.2	R Foundation for Statistical Computing	https://www.r-project.org
QIIME2 (v2021.2)	Caporaso Lab, Northern Arizona University	https://qiime2.org
ImageJ	National Institutes of Health	https://imagej.net/ij
FlowJo 10.0	BD Biosciences	https://www.flowjo.com
MetaboAnalyst 6.0	McGill University, Montreal, QC, Canada	https://www.metaboanalyst.ca
Gene Ontology	The Gene Ontology Consortium	https://geneontology.org/
Photoshop	Adobe	https://www.adobe.com/products/photoshop
GraphPad Prism	Software Inc.	https://www.graphpad.com/

**Other**		

NanoDrop ND-1000/2000	Thermo Fisher Scientific	https://www.thermofisher.cn
Qubit 2.0/3.0 Fluorometer	Thermo Fisher Scientific	https://www.thermofisher.cn
Illumina MiSeq System	Illumina	https://www.illumina.com/
Illumina NovaSeq 6000	Illumina	https://www.illumina.com/
Agilent 7890B GC-5977B MS	Agilent Technologies	https://www.agilent.com.cn
Agilent 1290 UPLC-6470 MS	Agilent Technologies	https://www.agilent.com.cn
BD FACSymphony™ A3 flow cytometer	BD Biosciences	https://www.bdbiosciences.com

### Experimental model and study participant details

#### Study design and participants

This study adopted a prospective, nested case-control design within the ongoing “Early Life Plan” birth cohort at Xinhua Hospital, Affiliated to Shanghai Jiaotong University School of Medicine.^43^ The study protocol was approved by the Ethics Committee of Xinhua Hospital (approval number: XHEC-C-2016-016), and written informed consent was obtained from all participating infants’ parents or legal guardians prior to enrollment.

Preterm infants meeting the inclusion criteria were recruited from the aforementioned cohort between January 2017 and December 2018. The inclusion criteria were as follows: (a) gestational age ≤32 weeks; (b) admitted to or referred to our hospital on the first day of life; (c) parents or legal guardians agreed to provide biospecimens (including blood and stool urine) during hospitalization and subsequent follow-up visits. Infants were excluded if they had congenital intestinal anomalies, major malformations, or a history of probiotic use (to minimize potential confounding effects on gut microbiota composition).

A total of 63 preterm infants were enrolled from the cohort of 294 infants with a gestational age ≤32 weeks. The study comprised 30 cases with BPD and 33 controls without BPD. The cases for BPD were defined as treatment with oxygen >21% for at least 28 days and the need for supplemental oxygen or positive pressure therapy at 36 weeks postmenstrual age. In this study, we enrolled infants of BPD meeting thresholds for moderate (oxygen requirement at 36 weeks’ postmenstrual age) and severe (need for ≥30% oxygen/positive pressure ventilation at 36 weeks’ postmenstrual age) disease phenotypes according the 2001 NICHD consensus criteria for BPD classification.^44^ Control subjects were preterm infants without BPD (required oxygen therapy less than 28 days).

All study participants were of Chinese ethnicity. Key demographic and clinical characteristics were collected, including gestational age (≤32 weeks), birth weight, and sex. Perinatal and demographic attributes, which were comparable between the BPD and control groups, are summarized in the main text (Table 1). The analysis confirmed that the groups did not have significant differences in sex, gestational age, or birth weight (all *p* ≥ 0.05). While the study collected data on sex, and its distribution was balanced between groups, the influence of sex on the primary outcomes (microbiome and metabolite profiles) was not the focus of this analysis; however, sex was included as a covariate in adjusted statistical models to control for its potential association with the results.

#### Mice

Wild-type pregnant Sprague-Dawley (SD) rats were obtained from the Shanghai Laboratory Animal Center (Shanghai, China). All animals were housed under specific pathogen-free (SPF) conditions in a temperature- and humidity-controlled room (23°C, 65% humidity) with a 12-h light/dark cycle and had free access to food and water. All experimental procedures were approved by the Animal Care and Use Committee of Xinhua Hospital, Shanghai, China (Approval No. XHEC-F-NSFC-2018-184) and were conducted in strict accordance with the institutional guidelines for the care and use of laboratory animals.

#### Creation of a rat model of BPD

To mechanistically explore the therapeutic potential of gut microbiota-associated metabolites (specifically cholic acids) in BPD, we used a prenatal chorioamnionitis-induced BPD rodent model. This model effectively mimics key pathophysiological features of human BPD, including alveolar simplification and pulmonary vascular remodeling.^45^

Briefly, intra-amniotic injections were administered on gestational day 16.5. Pregnant rats received either 5 μL of normal saline (control group) or 1 μg of lipopolysaccharide (LPS group) per amniotic sac, respectively. The day of pup delivery was designated as postnatal day 0 (P0). After delivery, building on the established therapeutic efficacy of bile acid interventions (e.g., 100 mg/kg chenodeoxycholic acid via feed),^46^ chorioamnionitis-induced BPD rat pups and saline control pups were randomly allocated to receive either cholic acid (CA, 100 mg/kg) or an equal volume of normal saline via daily orogastric gavage from P0 to postnatal day 6 (P6) using a 1.9 Fr single-lumen PICC catheter (10 cm in length). All animals were euthanized on postnatal day 7 (P7) for subsequent analyses. Due to the extremely early developmental stage of the pups (postnatal day 0–7), reliable sex identification based on external physical characteristics was not feasible.

#### Cell lines

The murine macrophage cell line RAW264.7 (TIB-71) cells and human HEK293T cells (CRL-3216) were purchased from the American Type Culture Collection (ATCC) and cultured in DMEM (10% FBS, 100 U/mL penicillin, 100 μg/mL streptomycin) at 37 °C, 5% CO_2_. The cells were stored in liquid nitrogen. Routine monitoring for mycoplasma contamination was performed using PCR-based assays.

Bone marrow-derived macrophages (BMDMs) were obtained from the femurs of 6-week-old Sprague-Dawley (SD) rats. The bone marrow cavities were flushed with sterile PBS to collect the marrow cells, which were then cultured in RPMI-1640 medium supplemented with 10% FBS, 10 ng/mL GM-CSF, and 1% penicillin/streptomycin. The cells were incubated at 37 °C in a 5% CO_2_ atmosphere for 7 days to allow differentiation into macrophages, with the culture medium refreshed every 2–3 days. After 7 days, the adherent BMDMs were harvested and used for subsequent experiments.

### Method details

#### Human sample collection

After enrollment, two fecal samples (5g/each) were collected from the nappies of each infant by means of a sterile swab and put in a sterile collection box without any preservation solution at the 36 weeks of postmenstrual age, and then quickly transported on ice to the hospital laboratory and frozen at −80 °C. Given that microbiota-derived metabolites can traverse the intestinal mucosa and contribute to pulmonary immunopathology through systemic circulation,^47^ the serum metabolite subcohort was randomly sampled from the total study cohort and 1:1 matched for key baseline characteristics, namely gestational age (±1 week) and birth weight (±100 g). Serum samples (500ul/each) and peripheral blood samples (2mL/each) were collected, processed and stored at −80 °C.

#### Fecal DNA extraction and 16S rRNA gene sequencing

Microbial genomic DNA was extracted from ∼0.25 g feces using the QIAamp PowerFecal Kit (Qiagen) with minor modification: samples were heated at 95 °C for 15 min instead of 60 °C, followed by homogenization with a Tissue-Lyser II (Qiagen). DNA concentration and purity were assessed using NanoDrop ND-1000 and Qubit 2.0 fluorometer with a high-sensitivity kit.

The V1-V3 region of 16S rRNA was amplified with primers 27F/534R using Phusion High-Fidelity PCR Mastermix (Invitrogen). Cycling: 95 °C 2 min; 30 cycles of 95 °C 20 s, 56 °C 30 s, 72 °C 1 min. Products were gel-verified, purified with AMPure XP beads (Beckman Coulter), and pooled equimolarly. Sequencing was performed on Illumina MiSeq with v3 600-cycle chemistry (2 × 300 bp), yielding ∼15,000 reads/sample. Raw reads were filtered, trimmed, and processed in QIIME2 (2021.2). Sequences were denoised with DADA2, chimeras removed, and samples rarefied to 15,000 reads. Taxonomic assignment, alpha/beta diversity, and differential abundance analysis (LEfSe, LDA score>3) were performed using QIIME2, PhyloSeq, and R packages microbiomeSeq and stats.

#### Fecal and serum metabolomics

For bile acid (BA) and short-chain fatty acid (SCFA) measurements, frozen feces and serum samples were shipped on dry ice to Shanghai Metabolome Institute-Wuhan (Wuhan, China).

SCFA quantification was performed by gas chromatography-mass spectrometry (GC-MS) following a previously described method.^48^ Fecal samples (approximately 20 mg) were placed in 2 mL Eppendorf tubes, mixed with 1 mL of 10% isobutanol, and homogenized using a TissueLyser (QIAGEN). After centrifugation (21,000 × g, 5 min), the supernatant was transferred to a clean tube. NaOH solution and chloroform were added, vortexed, and centrifuged (21,000 × g, 2 min). The supernatant was collected, and derivatization was performed by sequentially adding isobutanol, pyridine, and ultra-pure water, followed by careful addition of chloroformate. Finally, n-hexane was added, and after centrifugation, the upper layer was transferred to a GC vial.

For serum SCFA analysis, 200 μL of serum was mixed with 5 μL of internal standard (500 μmol/L propanoic acid-D_2_ in isobutanol), 40 μL isobutanol, 65 μL NaOH (20 mM), and 50 μL pyridine. Derivatization was carried out with 25 μL menthyl chloroformate. After adding 75 μL hexane, the upper hexane-isobutanol phase was transferred to a GC vial.

GC-MS analysis was conducted on an Agilent 7890B GC coupled to an Agilent 5977B MS (Agilent Technologies, USA). Separation was achieved on an HP-5MS column (0.25 μm × 0.25 mm × 30 m; 19091S-433, Agilent). Data acquisition and preprocessing were performed using MassHunter Workstation software (version B.08.00; Agilent). SCFAs were identified by comparing retention times and mass spectra with authentic standards. Quantification was based on standard curves constructed by linear regression of peak area ratios (analyte/internal standard) against nominal calibrator concentrations.

BA quantification was performed using ultra-performance liquid chromatography-tandem mass spectrometry (UPLC-MS/MS) as previously described.^49^ A mixture of 32 BA standards was prepared as internal standard. Fecal samples (10 mg) were combined with methanol/water (1:1, v/v) and internal standards, subjected to three rapid freeze-thaw cycles in liquid nitrogen, and homogenized using a TissueLyser. After centrifugation, the supernatant was transferred to an LC-MS vial.

For serum BA analysis, 100 μL of serum was mixed with 120 μL of internal standard (100 nmol/L CDCA-D_4_ and 100 nmol/L CA-D_4_ in methanol:water (2:1, v/v)) and 980 μL of pre-cooled methanol for protein precipitation. Following centrifugation (12,000 rpm, 4 °C, 10 min), the supernatant was evaporated to dryness and reconstituted in 50 μL of methanol:water (2:1, v/v) containing 0.005% formic acid, then filtered through a 0.22 μm membrane prior to analysis.

UPLC-MS/MS analysis was performed on an Agilent 1290 UPLC system coupled to an Agilent 6470 triple quadrupole mass spectrometer equipped with an electrospray ionization (ESI) source (Agilent Technologies, USA). Separation was carried out on an Agilent ZORBAX Eclipse Plus C18 column (2.1 × 100 mm, 1.8 μm). Data acquisition and analysis were performed using MassHunter Workstation software (version B.08.00). Quantification was performed using standard curves as described for SCFAs.

#### RNA-seq of human whole blood

TPeripheral blood (2 mL) was collected from 9 infants with BPD and 9 preterm control infants. Total RNA was extracted using TRIzol reagent (Invitrogen Life Technologies, Carlsbad, CA, USA) following the manufacturer’s instructions, and samples were submitted to Novogene Bioinformatics Institute (Beijing, China) for library construction and high-throughput sequencing.

RNA integrity was evaluated using the RNA Nano 6000 Assay Kit with an Agilent 2100 Bioanalyzer (Agilent Technologies, Santa Clara, CA, USA). For each library, 1 μg of total RNA was used for sample preparation. Messenger RNA was purified from total RNA using poly-T oligo-attached magnetic beads and fragmented with divalent cations at elevated temperature in 5× First Strand Synthesis Reaction Buffer. First- and second-strand cDNA was synthesized using random hexamer primers, M-MuLV Reverse Transcriptase (RNase H^−^), DNA Polymerase I, and RNase H. Remaining overhangs were converted into blunt ends using exonuclease/polymerase activities. After 3′-end adenylation, hairpin loop-structured adaptors were ligated for subsequent hybridization. cDNA fragments of 370–420 bp were size-selected using the Agencourt AMPure XP system (Beckman Coulter, Beverly, MA, USA). PCR amplification was performed using Phusion High-Fidelity DNA polymerase, universal PCR primers, and Index (X) primers. PCR products were purified using the AMPure XP system, and library quality was verified using the Agilent 2100 Bioanalyzer.

Index-coded samples were clustered on a cBot Cluster Generation System using the TruSeq PE Cluster Kit v3-cBot-HS (Illumina) according to the manufacturer’s protocol. After cluster generation, libraries were sequenced on an Illumina NovaSeq platform, and 150-bp paired-end reads were generated for downstream bioinformatic analysis.

#### Lung morphometry and immunostaining

Five to seven pups at P7 were selected from each group, and six random non-overlapping fields in one distal lung section per pup were utilized for morphometric examinations. The sample of left lungs were fixated in 4% paraformaldehyde, and were dehydrated in an increasing gradient of ethanol, cleared in xylene and embedded in histology paraffin. Histologic sections of 5 μm thickness were performed and stained with hematoxylin-eosin (H/E). Terminal airspace, secondary septa and mean linear intercept in each field were manually quantified.

The paraffin-embedded lung sample were placed in a repair box filled with citric acid antigen repair buffer, and antigen repair was conducted using the microwave thermal repair method. The lung tissue sections were immunostained with F4/80 (macrophages), iNOS (M1-like), and CD206 (M2-like).

#### Macrophage isolation, migration, and flow cytometry

Rat BMDMs were isolated from the femur and matured in RPMI 1640 medium supplemented with 10% fetal bovine serum, 10 ng/mL granulocyte-macrophage colony-stimulating factor, 100 U/ml penicillin, and 100 mg/mL streptomycin for 7 days. BMDMs were cultured with or without LPS (100 ng/mL) for 24 h, and then were stained with CFSE (5 mM). We injected 1 × 10^6^ cells of each population into neonatal rats via intraperitoneal route in three different groups (control, BPD model, and BPD with CA feeding model). We euthanized host rats 24 h later and determined the numbers of stained BMDMs in the lungs by flow cytometry in order to check their migration.

The peripheral lung tissue was cut into small pieces with scissors, and processed in digestion buffer, which contain 5 mL of RPMI1640 and 10%FBS, 5ul/ml Type VIII Collagenase and 1 μL/mL DNase I. Then the homogenized lungs were passed through 40-μm nylon mesh and was added percoll solution to obtain a single-cell suspension for further flow cytometry. Besides lung cell suspension, RAW264.7 cells (1×10^6^) were pretreated with CA (20 μM) for 2 h. Subsequently, the cells were stimulated with 100 ng/mL LPS for 24h.

All cells were stained for 1h at room temperature, and then washed in FACS buffer three times. Flow cytometry was performed on a BD FACSymphony A3 using antibodies against F4/80 and iNOS. Data were analyzed using FlowJo software.

#### Western blot

A piece of lung tissue of the right lobe of rats (50 mg) was obtained and grinded into powders. RAW264.7 cells (1×10^6^) were cultured with CA (20 μM) for 2 h, followed by stimulation with LPS at a concentration of 1 ng/mL for 24 h.

All the protein samples were extracted using RIPA buffer containing HALT protease and phosphatase inhibitor cocktails and subjected to electrophoresis by SDS-PAGE method and then transferred to PVDF membrane for further immunoblot. Blots were probed with antibodies against CCR5, HIF-1α, TGR5, FXR, and Tubulin. Signals were visualized using chemiluminescence. Adjust the exposure time according to the signal intensity to obtain clear bands.

#### ELISA

Levels of IL-1β, CCL3, CCL4, CCL5, and TNF-α in lung homogenates or RAW264.7 supernatants were measured using commercial ELISA kits (R&D Systems, MultiSciences, Bosterbio, ELK Biotechnology) according to manufacturer protocols.

#### RT-qPCR

HIF-1α mRNA levels were assessed by RT-qPCR. Briefly, total RNA was extracted with TRIeasy Total RNAExtraction Reagent(TCM Free) and was reverse-transcribed to cDNA by Hifair AdvanceFast 1st Strand cDNA Synthesis Kit (Yeasen). qPCR was run Hieff UNICON Universal Blue qPCR SYBR Green Master Mix (Yeasen) and primers targeting HIF1α (HIF-1α-F:GGTATTATTCAGCACGACTT, HIF-1α-R:GGAGACATTGCCAGGTTTAT) and the housekeeping gene ACTB. Amplification involved 40 cycles (95 °C for 10 s, 60 °C for 30 s) after an initial 95 °C step for 2min. Specificity was confirmed by melt curve analysis, and relative expression was determined via the 2^−^ΔΔCT method.

#### BALF bulk RNA sequencing

BALF samples were centrifuged at 300×g for 10 min to pellet cells. Total RNA was then extracted from the cell pellet using TRIzol reagent. RNA quality was determined using the ratio of A260/A280 with a Nanodrop 2000 spectrophotometer. RNA samples were further tested using an Agilent 2100 Bioanalyzer (Agilent Technologies, USA) in the Institution of Immunology, Shanghai Jiaotong University School of Medicine.

RNA integrity was further eveluated using an Agilent 2100 Bioanalyzer (Agilent Technologies,USA). Samples with RIN>7 were selected for sequencing library construction using the Illumina Stranded mRNA Prep, Ligation kit (Illumina, USA). The final library was sequenced using an Illumina Novaseq 6000 sequencer. On average, about 20 million 150-bp paired end reads were generated per sample.

Using HISAT2 RNA-sequencing alignment software, the raw reads were quality-checked (Q30 > 85%) and aligned to the mRatBN7.2 genome using HISAT2. The mapping rate was 96%, on average, across all the samples in the dataset. The alignment files (bam files) were processed to generate read counts for each gene using HISAT and HTSeq. The read counts were normalized using the R package DESeq2 and then were centered and scaled for each gene to generate z-scores. Genes with a Benjamini–Hochberg adjusted *p*-value <0.05 and an absolute fold change ≥2 were considered statistically significant. Gene ontology (GO) and Kyoto Encyclopedia of Genes and Genomes (KEGG) analyses were performed using OmicsBean software.

#### siRNA transfection and luciferase assay

HIF-1α siRNA was purchased from GenePharma Co., Ltd. (Shanghai, China). The RAW 264.7 were seeded into a 24-well plate. When cell confluence reached 70%–90%, the cells were then transfected in accordance with the Lipofectamine 3000 operational instructions. Briefly, the lipofectamine 3000 and siRNA plasmids was mixed and diluted in Opti-MEM according to the instructions provided. Mix the siRNA and lipo3000 reagent and then added to the culture plate for a 15-min period of incubation in dark. The culture medium was then replaced with complete culture medium. After 24–48 h, the transfected cells were subjected to a cell-based perfusion assay. The cells were subsequently assigned into four groups according to the siRNA sequencing, namely S1-S4 groups. Then, knockdown efficiency was validated by immunoblot analysis.

CA binding sites in the HIF-1α promoter region was predicted using an online-based prediction tools available at RCSB PDB (https://www.rcsb.org/). HIF-Luc plasmid was generated by PCR amplification from the cloned pGL3-basic vector (−341 bp to −345 bp (GCGCA)). Then HIF-Luc plasmid was cotransfected into HEK293T cells as previous studies.^50^ After transfection, the cells were treated with CA (50 μg/L) for 24h. Then Luciferase activity of cell lysates was determined luminometrically by the dual-luciferase assay system (Promega) as specified by the manufacturer. Each transfection was performed in triplicate, and all experiments were repeated for nine times.

### Quantification and statistical analysis

Statistical analysis was performed using IBM SPSS software, version 20.0 (SPSS, Inc., Chicago, IL, USA) or R version 4.0.2. Chi-square (χ2) or Fisher’s exact test was used for comparisons of categorical variables, and Student’s *t* test (normally distributed data) or Mann-Whitney test (not-normally distributed data) for comparisons of continuous variables. Data were expressed as mean ± standard deviation (SD), median (interquartile range, IQR), or number (percentage). Multivariable analysis was performed using a generalized linear model to control the potential confounders.

The alpha and beta diversity of the microbiota was assessed in R. Linear discriminant analysis effect size analysis was performed to identify differential microbiota between BPD and control groups, with the threshold set at LDA score >3. For multiple hypothesis testing in microbiome and metabolomics analyses, the False Discovery Rate (FDR) was controlled using the Benjamini-Hochberg method. An FDR-adjusted *p*-value of less than 0.05 was considered statistically significant.

Pathway analysis for the differential metabolites was conducted using the MetaboAnalyst platform. For the differential genes, Gene Ontology (GO) analysis and visualization were performed using the Metascape platform. A *p*-value of <0.05 was considered to be statistically significant.
