# Supplementary material for: Gut microbiota-derived cholic acid ameliorates lung inflammation in bronchopulmonary dysplasia through modulation of macrophage function
Source: iScience. 2026 Mar 17;29(4):115398. doi: 10.1016/j.isci.2026.115398 (PMC13068612; doi:10.1016/j.isci.2026.115398)

iScience, Volume *29*

Supplemental information

Gut microbiota-derived cholic acid ameliorates lung inflammation in bronchopulmonary dysplasia through modulation of macrophage function

Dongying Zhao, Caixia Gao, Danying Zhu, Xiaoyan Zheng, Jiping Sun, Chengbo Liu, Lei Chen, Lei Shen, Xingyun Wang, and Yongjun Zhang

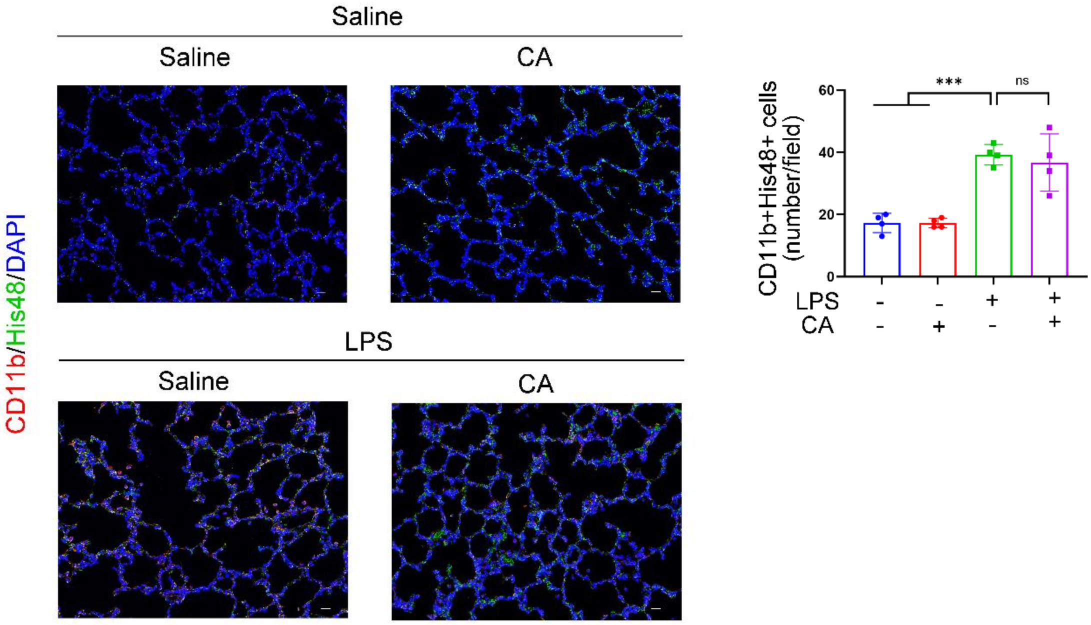

Supplementary Figure S1. Effects of cholic acid on neutrophil accumulation in lung.

Microscopy images of left lung tissues from chorioamnionitis-induced bronchopulmonary dysplasia (BPD) model rats or saline-treated control rats, following oral gavage with cholic acid (CA) or saline for 7 days. Lung sections were immunostained for the neutrophil markers CD11b and His48. Scale bar: 20 µm. Representative fields were analyzed to quantify CD11b$^+$His48$^+$ double-positive cells. Data are expressed as mean ± SD (n=4). * p<0.05; **, p<0.01; ***, p<0.001.

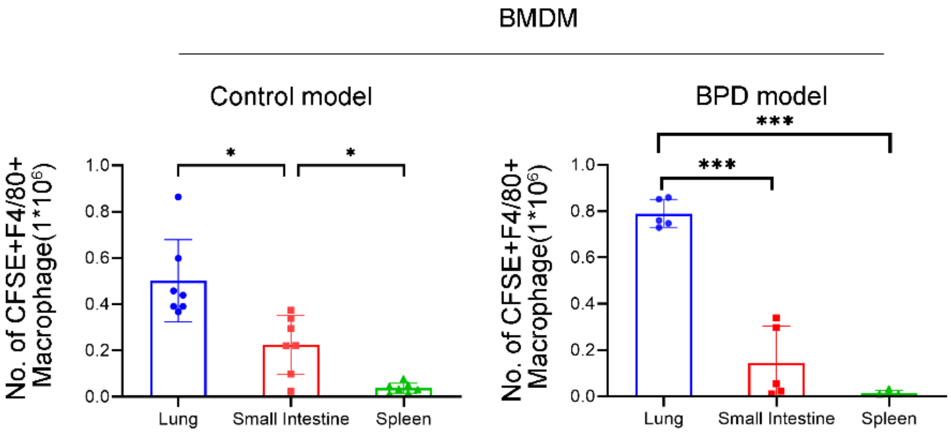

Supplementary Figure S2. Migration of bone marrow-derived macrophages to the lungs in bronchopulmonary dysplasia rats.

Rat BMDMs were isolated and cultured. After BMDMs were stained with CFSE, it was injected into neonatal rats via intraperitoneal route in two different groups (control, BPD model) for 24 hours. Quantitative analysis of the number of CFSE-stained F4/80 macrophage in the rat lungs, small intestine and spleen. In BPD model, almost all the labeled macrophages were transfer to the lung instead of small intestine and spleen. Data are expressed as mean ± SD (*n*=5-7). * $p<0.05$; **, $p<0.01$; ***, $p<0.001$.

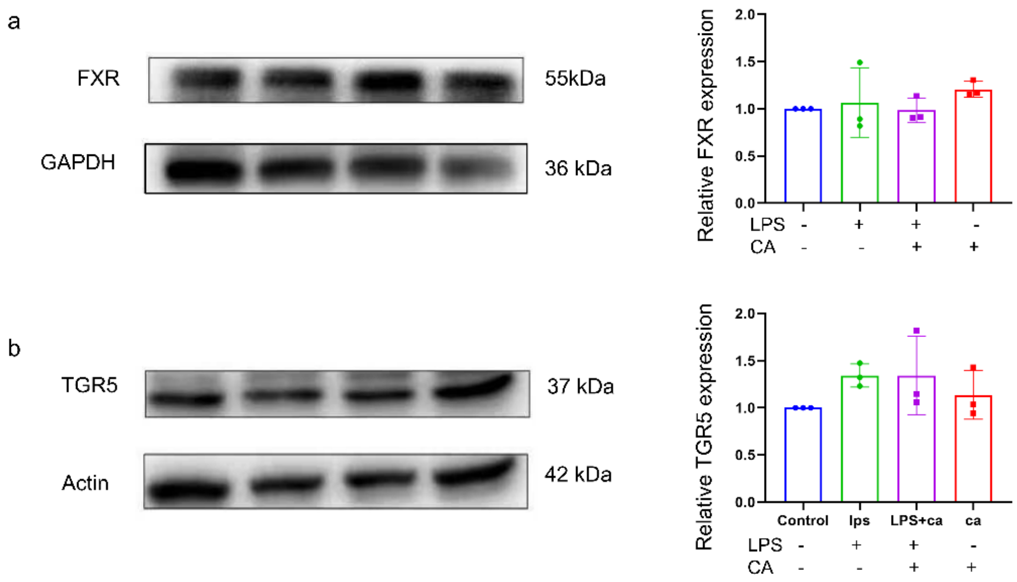

Supplementary Figure S3. Effects of cholic acid on bile acid receptor in lung.

Chorioamnionitis-induced BPD rats and saline controls were fed CA or saline for 7 days (n=3/group) to assess lung bile acid receptor signaling. Representative immunoblots and relative FXR (a) and TGR5 (b) expression were detected in P6 rat whole lung lysates. All data are presented as mean ± SD (n=3 per group).

Supplementary Table S1. List of 15 short-chain fatty zcids and 32 bile acids standard compounds and their LOQ obtained from our study

Abbreviation	Name	LOQ（fmol）
Short-Chain Fatty Acids		
	Formic acid	236.73
	Acetic acid	446.26
	Propanoic acid	73.53
	Isobutyric acid	1236.38
	Butyric acid	24.72
	2-Methylbutyric acid	10.67
	Isovaleric acid	10.67
	Valeric acid	10.67
	3-Methylpentanoic acid	18.75
	Isocaproic acid	18.57
	Hexanoic acid	9.39
	2-Methylhexanoic acid	8.37
	4-Methylhexanoic acid	16.73
	Heptanoic acid	16.73
	Octanoic acid	7.56
Bile Acids		
Taurine-conjugated		
THCA	Taurohyocholic acid	5.09
T-α-MCA	Tauro-alpha-muricholic acid	5.81
T-β-MCA	Tauro-beta-muricholic acid	7.63
TCDCA	Taurochenodeoxycholic acid	6.45
TCA	Taurocholic acid	20.45
TDCA	Taurodeoxycholic acid	5.47
TUDCA	Tauroursodeoxycholic acid	2.11
TLCA	Taurolithocholic acid	3.03
THDCA	Taurohyodeoxycholic acid	1.51
Glycine-conjugated		
GCA	Glycocholic acid	3.88
GHDCA	Glycohyodeoxycholic acid	1.81

GCDCA	Glycochenodeoxycholic acid	2.48
GDCA	Glycodeoxycholic acid	4.78
GUDCA	Glycoursodeoxycholic acid	1.57
GLCA	Glycolithocholic acid	1.29
GHCA	Glycohyocholic acid	1.57
Unconjugated		
CA	Cholic acid	5.02
HCA	Hyocholic acid	3.70
α-MCA	Alpha-muricholic acid	3.47
β-MCA	Beta-muricholic acid	1.85
ACA	Allocholic acid	7.41
CDCA	Chenodeoxycholic acid	2.18
DCA	Deoxycholic acid	2.86
iso-DCA	Isodeoxycholic acid	3.23
HDCA	Hyodeoxycholic acid	2.24
UDCA	Ursodeoxycholic acid	2.92
nutriCA	Nutriacholic acid	2.00
12-ketoDCA	12-Ketodeoxycholic acid	3.25
LCA	Lithocholic acid	2.27
iso-LCA	Isolithocholic acid	6.74
ω-MCA	Omega-muricholic acid	2.99
MDCA	Murideoxycholic acid	1.74

LOQ, Limit of quantitation

Supplementary Table S2. Short-chain fatty acids and bile acids in fecal sample of BPD patients and preterm controls

**Metabolites**	**BPD group** (n=30)	**Control group** (n=33)	***p* value**
Formic acid (μg/g)	9.69 (2.33, 37.52)	8.78 (2.98, 106.09)	0.785
Acetic Acid (μg/g)	2018.38 (559.42, 3564.45)	2367.64 (1223.72, 6210.57)	0.137
Propanoic acid (μg/g)	68.60 (22.96, 124.35)	415.91 (59.56,1105.33)	**0.005**
Butyric acid (μg/g)	98.93 (39.65, 396.83)	559.72 (50.25, 891.71)	0.138
2-Methylbutyric acid (μg/g)	13.91 (1.20,55.10)	41.99 (21.65, 116.94)	0.169
Isovaleric acid (μg/g)	10.40 (1.43, 51.14)	27.09 (6.86, 110.06)	0.627
Valeric acid (μg/g)	3.14 (1.53, 5.19)	10.18 (2.71, 31.19)	**0.031**
Isocaproic acid (μg/g)	17.66 (6.60, 85.45)	44.31 (31.16, 90.85)	0.061
Hexanoic acid (μg/g)	0.91 (0.62, 2.05)	1.41 (0.87, 3.36)	0.161
Octanoic acid (μg/g)	5.94 (1.79,13.28)	11.37 (6.56, 26.27)	0.320
THCA (nmoL/g)	23.75 (4.00, 73.45)	32.56 (4.32, 134.05)	0.560
TCDCA (nmoL/g)	94.93 (24.34, 224.83)	25.31 (8.48, 515.15)	0.346
TCA (nmoL/g)	53.80 (15.09, 280.99)	23.31 (7.19, 1164.45)	0.845
GCA (nmoL/g)	6.48 (1.71, 24.90)	5.88 (1.98, 59.97)	0.529
GCDCA (nmoL/g)	2.29 (0.40, 10.61)	0.99 (0.38, 20.98)	0.924
CA (nmoL/g)	125.71 (12.53, 669.84)	755.28 (155.21, 2146.54)	**0.002**
HCA (nmoL/g)	5.42 (1.18, 31.46)	44.56 (12.43, 70.93)	**0.006**
CDCA (nmoL/g)	86.98 (7.24, 512.48)	412.64 (107.10, 1017.18)	**0.011**
DCA (nmoL/g)	0.63 (0.06, 1.05)	0.98 (0.75, 1.88)	0.798
UDCA (nmoL/g)	3.36 (0.96, 33.49)	8.71 (1.04, 98.12)	0.664
nutriCA (nmoL/g)	14.22 (3.78, 107.42)	43.52 (6.33,120.14)	0.341

Data are presented as median (IQR)

THCA, taurohyocholic acid; TCDCA, taurochenodeoxycholic acid; TCA, taurocholic acid; GCA, glycocholic acid; GCDCA, glycochenodeoxycholic acid; CA, cholic acid; HCA, hyocholic acid; CDCA, chenodeoxycholic acid; DCA, deoxycholic acid; UDCA, ursodeoxycholic acid; nutriCA, nutriacholic acid

Supplementary Table S3. Generalized linear model analysis of metabolites associated with BPD in fecal samples (n=63)

Metabolites*	Odds Ratio	95% Confidence Interval	*p* value	Adjusted Odds Ratio**	95% Confidence Interval	*p* value
Cholic acid	0.836	0.829, 0.748	**0.002**	0.841	0.766, 0.923	**<0.001**
Chenodeoxycholic acid	0.836	0.742, 0.943	**0.004**	0.873	0.786, 0.970	**0.012**
Hyodeoxycholic acid	0.830	0.735, 0.937	**0.003**	0.836	0.755, 0.925	**0.001**
Propanoic acid	0.795	0.685, 0.923	**0.003**	0.777	0.678, 0.890	**<0.001**
Valeric acid	0.709	0.541, 0.929	**0.013**	0.716	0.543, 0.945	**0.018**

*Non-normally distributed metabolites were log-transformed.

**Adjusted for gestational age, birth weight, sex, mode of feeding, duration of mechanical ventilation, duration of CPAP, and days of antibiotic use by using generalized linear model

Supplementary Table S4. Demographic characteristics of the selected and non-selected infants. (N = 63)

Parameters	Selected infants n=24	Non-Selected infants n=39	*p* value
Demographic data			
Birth weight (g)	1137.7±196.6	1085.0±214.9	0.333
Gestational age (wk)	28.1±1.8	27.6±1.8	0.309
Sex			0.735
Male	12	23	0.486
Female	12	16	
Mode of delivery			
Vaginal	13	18	0.537
Cesarean	11	21	

Data are presented as mean ± S.D. or N.

Supplementary Table S5. Demographic characteristics of the infants underwent serum metabolites measurement. (N = 24)

Parameters	BPD<br>n=12	Control<br>n=12	*p* value
Demographic data			
Birth weight (g)	1075.83±109.9	1199.58±245.8	0.126
Gestational age (wk)	27.9±1.8	28.3±1.9	0.592
Sex			0.735
Male	6	6	1.000
Female	6	6	
Mode of delivery			
Vaginal	6	5	0.682
Cesarean	6	7	

Data are presented as mean ± S.D. or N.

Supplementary Table S6. Generalized linear model analysis of metabolites associated with BPD in serum samples (n=24)

**Metabolites[*]**	**Odds Ratio**	**95% Confidence Interval**	***p* value**	**Adjusted Odds Ratio[**]**	**95% Confidence Interval**	***p* value**
Cholic acid	0.658	0.457, 0.947	**0.024**	0.724	0.520, 1.008	0.056
Chenodeoxycholic acid	0.911	0.621, 5.884	0.794	0.865	0.472, 1.585	0.639
Propanoic acid	0.514	0.204, 1.296	0.158	0. 739	0.297, 1.834	0.514

*Non-normally distributed metabolites were log-transformed.

** Adjusted for gestational age, birth weight, sex, mode of feeding, duration of mechanical ventilation, duration of CPAP, and days of antibiotic use by using generalized linear model

Supplementary Table S7. Demographic characteristics of the infants underwent whole blood sequencing (N = 18)

**Parameters**	**BPD**<br>n=9	**Control**<br>n=9	***p* value**
Demographic data			
Birth weight (g)	1025.0±155.16	1062.2±212.1	0.677
Gestational age (wk)	27.7±1.4	28.1±1.7	0.855
Sex			0.735
Male	4	4	1.000
Female	5	5	
Mode of delivery			
Vaginal	4	6	0.343
Cesarean	5	3	

Data are presented as mean ± S.D. or N.

Supplementary Table S8. The sequence of HIF-1α-siRNA

Gene name	Sequence	
	sense（5'-3'）	antisense（5'-3'）
HIF-1α-S1	GAGCUUUGGAUCAAGUUAATT	UUAACUUGAUCCAAAGCUCTT
HIF-1α-S2	GCUGACCAGUUACGAUUGUTT	ACAAUCGUAACUGGUCAGCTT
HIF-1α-S3	CCACCACUGAUGAAUCAAATT	UUUGAUUCAUCAGUGGUGGTT
HIF-1α-S4	GAUGGAAGCACUAGACAAATT	UUUGUCUAGUGCUUCCAUCTT

Supplementary Data S1: Original protein bands

Fig 4f

CCR5

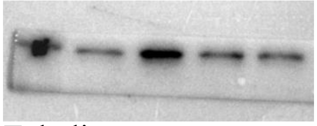

Tubulin

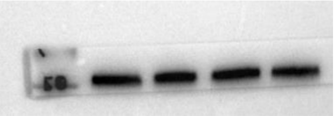

Fig 5a

CCR5

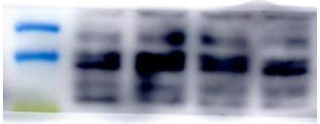

Tubulin

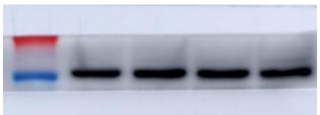

Fig 5g

HIF-1

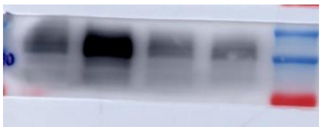

Tubulin

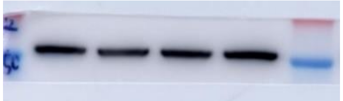

Fig 6c

HIF-1

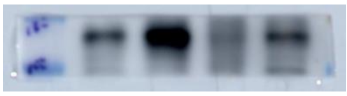

actin

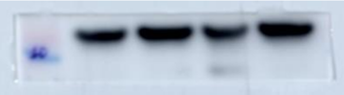

Fig 6 d

HIF-1

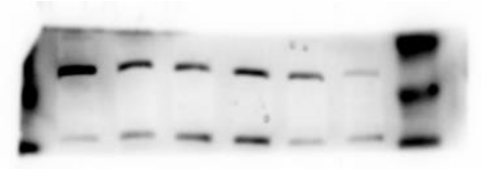

Tubulin

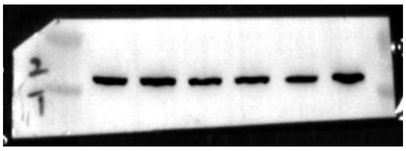

Fig 6e

HIF-1

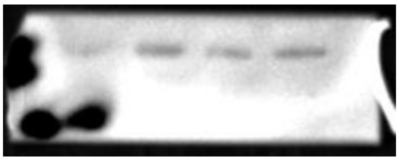

CCR5

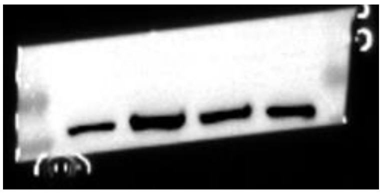

Tubulin

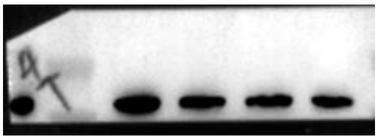

Supplemental Figure S3a. (right band)

FXR

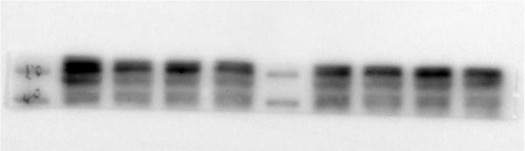

GAPDH

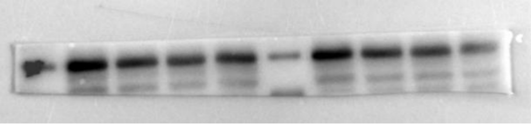

Supplemental Figure S3b. (right band)

Tgr5

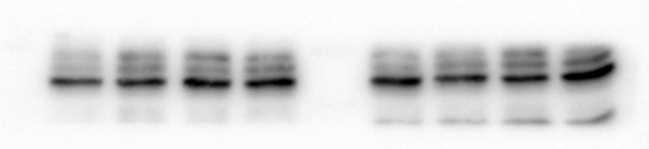

Actin

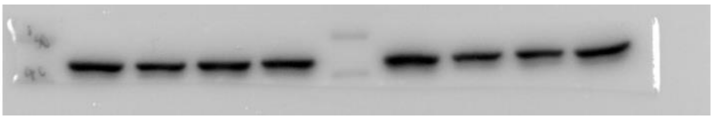

Supplement: Document S1. Figures S1–S3, Tables S1–S8, and Data S1 [file mmc1.pdf]
